# Prevalence, Themes, and Partisan Differences in US State Legislator X Posts Mentioning Suicide: Content Analysis

**DOI:** 10.2196/83018

**Published:** 2026-05-25

**Authors:** Michal Weiss, Madison Kitchen, Andrew Riblet, Katherine Keyes, Jonathan Purtle

**Affiliations:** 1 Department of Public Health Policy and Managment, School of Global Public Health, New York University, 708 Broadway, New York, NY, 10003, United States, 1 9148151697; 2 Silver School of Social Work, New York University, New York, NY, United States; 3 Department of Epidemiology, Mailman School of Public Health, Columbia University, New York, NY, United States

**Keywords:** suicide, content analysis, social media, state legislators, dissemination, United States, mental health

## Abstract

**Background:**

Suicide is a leading cause of death in the United States, and state policies can be effective tools to prevent suicide. State legislators are increasingly active on social media, communicating about their legislative priorities and signaling information about their knowledge and attitudes about issues.

**Objective:**

This study aimed to characterize US state legislators’ social media posts mentioning suicide on X (formerly Twitter) and explore differences in how Democrat and Republican legislators communicate about suicide.

**Methods:**

We used Quorum, a public affairs database, to identify all state legislator X posts mentioning suicide (N=1049) between December 1, 2023, and November 30, 2024. We developed a codebook and used content analysis to characterize posts and document the frequency of communication about suicide and themes related to causes, solutions, and consequences of suicide. We assessed concordance between the social media post language used and guidelines for reporting about suicide. We conducted univariate analysis and chi-square tests to assess differences in the content of posts between Democrat and Republican legislators. Differences in the frequency of posts about suicide were analyzed using 2-tailed *t* tests.

**Results:**

Of 1049 posts identified, 849 (80.9%) were included in the final sample. The annual suicide post rate per 10,000 posts was 13.2 (0.1% of all posts) among Democrats and 7.4 (0.1% of all posts) among Republicans (*P*=.09). Suicide related to a specific population was identified in 52.2% (443/849) of posts, with youth, veterans, firearm owners, and the LGBTQ+ (lesbian, gay, bisexual, transgender, queer, and more) population being identified most frequently. Causes of suicide were identified in 37.1% (315/849) of posts, with no significant difference between Democrats and Republicans. However, the types of causes identified varied, with Democrats more likely to identify lethal means (eg, firearms) as a cause of suicide than Republicans (115/573, 20.1% vs 20/172, 7.5%; *P*<.001). About two-thirds (558/849, 65.7%) of posts identified at least one solution to prevent suicide, with Democrats more likely to identify a solution than Republicans (443/573, 77.3% vs 114/268, 42.5%; *P*<.001). General awareness was the most frequent solution, while policy-specific solutions were present in only 23.3% (198/849) of posts. Collateral consequences of suicide were infrequently mentioned.

**Conclusions:**

This study found differences between Democrats and Republicans in their X posts about suicide and areas of misalignment with research evidence. When considered within the context of research on the epidemiology of suicide and evidence supporting suicide prevention policies, the study highlights the need to improve communication about suicide with state legislators and to encourage further collaboration with suicide prevention organizations and experts. Furthermore, given the differences observed, study findings suggest potential value in tailoring messages about suicide for legislators based on their political party.

## Introduction

Suicide is one of the leading causes of death in the United States and a major public health concern [1,2]. In 2023, more than 49,000 people died by suicide in the United States, an estimated 1.5 million attempted suicide, and an estimated 3.7 million made a suicide plan [3]. Suicide can be prevented through primary, secondary, and tertiary prevention strategies. Public policies can contribute to suicide prevention across this continuum by addressing structural determinants of suicide risk, promoting and providing access to mental health services, and limiting access to lethal means for completing suicide [4]. At the federal level in the United States, recent policy initiatives have targeted suicide prevention, such as the 988 Suicide and Crisis Lifeline and investments in Certified Community Behavioral Health Centers that are required to provide suicide prevention crisis services [5,6]. State-specific policies are also critical to US suicide prevention because most public health authority exists at the state level [7,8].

There are 7,386 state legislators in the United States, and many of these policymakers regularly use social media to communicate with the public about their legislative priorities and positions [9]. State legislators’ social media posts may shed light on how they think about various issues, highlight areas of misalignment between their public communications and research evidence, and identify partisan differences in policy discourse. This information can then be used to inform the design of policymaker-focused dissemination strategies. Legislators’ social media posts are thus increasingly analyzed as a data source in public health research [10-15].

Although suicide prevention has historically been considered an issue with bipartisan support [16], recent public opinion surveys suggest that Democrats and Republicans may have increasingly divergent views on suicide prevention interventions [17,18]. This polarization may extend to state legislators, as it has previously for other public policy topics [19-22]. For example, a previous study of state legislators’ social media posts about the 988 Suicide and Crisis Lifeline found that Democrats posted about 988 at a higher rate than Republicans [23]. However, to our knowledge, no prior studies have examined the content of state legislators’ public-facing communications about suicide more broadly.

To address this knowledge gap, this study aimed to characterize state legislators’ social media posts mentioning suicide on X (formerly Twitter). Specifically, the study aimed to document the incidence rate of suicide-related posts by state legislators, as well as the prevalence of themes about suicide-related causes, consequences, and solutions.

## Methods

### Ethical Considerations

The New York University Institutional Review Board deemed that the study was not considered human subjects research because the social media post data were publicly available (ie, from the public social media accounts of elected public officials). Therefore, further ethics approval was not required.

### Sample

This study used Quorum, a public affairs database, to identify all X posts by US state legislators that mentioned suicide between December 1, 2023, and November 30, 2024. Quorum is a web-based public affairs software program that centralizes, in near real-time, policymakers’ social media posts and other textual artifacts of policymaking processes [24]. We limited our analysis to a 1-year period given the exploratory, cross-sectional nature of the study and used the most recent data that were available when analyses began.

### Search Strategy and Sampling

Search terms were informed by extant literature [25] and developed by the study team to identify X posts that mentioned suicide and related terms. The search query used was: (“suicide” OR “suicidal” OR “suicide” OR “suicide prevention” OR “suicidal prevention” OR “suicidal ideation” OR “suicide ideation” OR “suicidality” OR “unalive” OR “unaliving” OR “killing oneself” OR “killing themselves” OR “offing” OR “sucide” OR “suicde” OR “unalive” OR “unaliving” OR “offing”). Reposts were excluded from the analysis to prevent the analytic sample from being skewed by the content of single posts that were reposted with high frequency.

### Codebook Development and Coding Process

Preliminary coding categories and definitions were developed based on studies related to the epidemiology of suicide policy, approaches to addressing suicide [4,26], and prior content analyses of textual news and social media data [27]. Following a review of a random sample of 50 posts by 3 study team members, the codebook was revised to update definitions and add emergent categories. This process was repeated 2 more times, with the 3 team members coding 25 posts each time, followed by codebook revisions.

The codebook included the following primary domains: characteristics of the legislator (ie, political party and state), the population that suicide was being mentioned in reference to (eg, youth and veterans), causes of suicide (eg, bullying and loneliness), solutions to prevent suicide (eg, restricting access to lethal means and increasing access to mental health services), and collateral consequences of suicide (eg, suicide contagion and bereavement). The study team also examined whether each X post was about an individual person, included statistics about suicide, cited a specific state bill about suicide, or encouraged self-help (eg, use of the 988 Lifeline). Posts were further coded according to whether they used language that was concordant with guidelines for reporting on suicide by Suicide Awareness Voices of Education (SAVE), which were collaboratively developed with input from organizations such as the American Foundation for Suicide Prevention and the US Substance Abuse and Mental Health Services Administration [28]. Examples of posts that were nonconcordant with these guidelines include those that mentioned “kill themselves” or “commit suicide,” or that made light of suicide or used the word suicide or related terms in a joking manner. The complete codebook is included in the Multimedia Appendix 1.

Three study team members coded posts using the finalized coding guide. Of 1,049 posts, a random sample of 200 (19.1%) were double coded. The mean interrater agreement was 91% (SD 11.2%) for binary (yes/no) coding categories and 88% (SD 7.6%) when including codes with multiple response options. The same 3 coders then independently coded the remaining 80.9% (n=849) of posts. This coding team met weekly to discuss any ambiguities or issues in the coding process and came to a consensus. Duplicate or near-duplicate posts identified throughout the coding process were removed from the sample to maintain consistency with the exclusion of reposts. Near-duplicate posts included instances in which a X post was uploaded a second time to correct grammar or spelling, add a hashtag, or include similar minor edits. Posts were coded using Qualtrics (Qualtrics, Inc), a web-based survey tool.

### Data Analysis

The final dataset of coded X posts was imported into SPSS (version 29.0.2.0; IBM Corp) for analysis. Univariate descriptive statistics were generated to characterize the prevalence of themes in the sample. The study team then stratified the sample according to the political party affiliation of the legislators, calculated the prevalence of themes among legislators in each party, and used chi-square tests to assess the statistical significance of differences in themes between Democrats and Republicans. Furthermore, to account for differences in the number of Democrat and Republican legislators and variability in the extent to which each party is active on X, Quorum was used to estimate the total number of posts by legislators of each party every month. Party-specific rates of suicide-related posts per 10,000 X posts were calculated. *t* tests were then used to assess statistical significance of differences between Democrat and Republican legislators, an approach that has been used in prior work [23].

Posts that primarily mentioned suicide in reference to war (eg, “suicide bombing”; 18/1049, 1.7% of posts) and physician-assisted suicide (88/1049, 9.3% of posts) were excluded from the analytic sample as we focused on self-directed harm only. More specifically, suicidal acts of terrorism were excluded because of the primary intent of harming others, and physician-assisted suicide was excluded because the death is not the result of self-directed harm but instead includes a lengthy process, approval, and a physician.

## Results

### Overview

**Figure 1.**
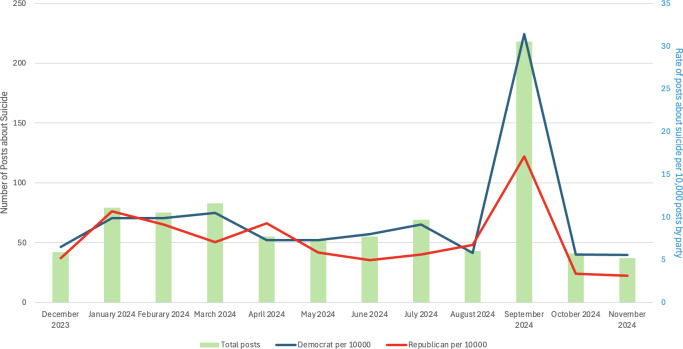
Volume of US state legislator X posts mentioning suicide.

Over the 1-year study period, state legislators posted 955 unique X posts mentioning suicide, and 849 (88.9%) posts were included in the analytic sample after removing posts primarily mentioning terrorism-related suicide and physician-assisted suicide. A total of 573 (67.5%) posts were from Democrats, 268 (31.6%) were from Republicans, and 8 (0.9%) were from Independents. The annual suicide tweet rate per 10,000 X posts was 13.2 (0.1% of all posts) among Democrats and 7.4 (0.1% of all posts) among Republicans (*P*=.09). Legislators from both parties posted the most about suicide in September, which is Suicide Prevention Awareness Month (Figure 1). When September was excluded from the analysis, there was a significant difference in the rate of suicide posts between Democrats and Republicans during the rest of the year (405/394,700, 0.001% vs 219/341,100, 0.001%; *P*=.003).

### Specific Populations Identified in Reference to Suicide

Of 849 posts, 443 (52.2%) mentioned a specific population in reference to suicide. The most frequently mentioned populations were youth (n=186, 21.9%), veterans (n=106, 12.5%), firearms owners or people with easy access to firearms (n=103, 12.1%), and LGBTQ+ (lesbian, gay, bisexual, transgender, queer, and more) individuals (n=82, 9.7%; Table 1). Democrats were significantly more likely than Republicans to mention youth (138/573, 24.1% vs 48/268, 17.9%; *P*=.04) and firearm owners or people with easy access to firearms (92/573, 16.1% vs 11/268, 4.1%; *P*<.001), while Republicans were significantly more likely to mention LGBTQ+ individuals (36/268, 13.4% vs 45/573, 7.9%; *P*=.04). First responders (n=13, 1.5%) were infrequently mentioned while older adults were never mentioned (n=0, 0%).

**Table 1.** State legislators’ X posts mentioning suicide from December 2023 to November 2024^a^.

Variable	Full sample (n=849), n (%)	Democrat posts (n=573), n (%)	Republican posts (n=268), n (%)	*t* test (df)	Chi-square (*df*)	*P* value
Any population specified in reference to suicide				—^b^	6.2 (1)	.01
No	406 (47.8)	255 (44.5)	144(53.7)			
Yes	443 (52.2)	318 (55.5)	124 (46.3)			
Specific population						
Children, teens, youth, and college students	186 (21.9)	138 (24.1)	48 (17.9)	2.01 (839)	—	.04
Disability or chronic illness	2 (0.2)	2 (0.3)	0 (0)	0.97 (839)	—	.33
Older adults	0 (0)	0 (0)	0 (0)	—	—	—
First responders	13 (1.5)	10 (1.7)	3 (1.1)	0.69 (839)	—	.49
Firearm owners or people with easy access to firearms	103 (12.1)	92 (16.1)	11 (4.1)	4.99 (839)	—	<.001
Health care providers	0 (0)	0 (0)	0 (0)		—	—
Incarcerated Individuals	2 (0.2)	1 (0.2)	1 (0.4)	−0.55 (839)	—	.58
LGBTQ+^c^ individuals	82 (9.7)	45 (7.9)	36 (13.4)	−2.56 (839)	—	.01
People who use drugs	7 (0.8)	2 (0.3)	5 (1.9)	−2.26 (839)	—	.02
People living with HIV	0 (0)	0 (0)	0 (0)	—	—	—
People living with mental illness	12 (1.4)	9 (1.6)	3 (1.1)	0.51 (839)	—	.61
Racial and ethnic minority groups	17 (2)	16 (2.8)	1 (0.4)	2.33 (839)	—	.02
Rural communities and farmers	12 (1.4)	6 (1)	6 (2.2)	−1.36 (839)	—	.18
Unhoused individuals	3 (0.4)	2 (0.3)	1 (0.4)	−0.06 (839)	—	.96
Veterans	106 (12.5)	75 (13.1)	31 (11.6)	0.619 (839)	—	.54
Posted about a specific person				—	3.868 (1)	.05
No	759 (90.1)^d^	524 (91.8)^e^	230 (87.5)^f^			
Yes	83 (9.9)^d^	47 (8.2)^e^	33 (12.5)^f^			
Included statistics				—	0.198 (1)	.66
No	640 (75.4)	428 (74.7)	204 (76.1)			
Yes	209 (24.6)	145 (25.3)	64 (23.9)			
Cited a specific bill				—	5.957 (1)	.02
No	665 (78.3)	434 (75.7)	223 (83.2)			
Yes	184 (21.7)	139 (24.3)	45 (16.8)			
Encouraged self-help				—	5.858 (1)	.02
No	692 (81.5)	454 (79.2)	231 (86.2)			
Yes	157 (18.5)	119 (20.8)	37 (13.8)			

a Eight X posts were excluded from the comparisons between Democrats and Republicans because they were posted by Independents, a group too small to analyze independently.

b Not applicable.

c LGBTQ+: Lesbian, gay, bisexual, transgender, queer, and more.

d n=842.

e n=571.

f n=263.

### Factors Identified as Causes of Suicide

Of 849 posts, 315 (37.1%) identified at least one factor as a cause of suicide, with no significant difference in the prevalence of a cause identified between Democrats and Republicans. The most frequent causes for suicide identified were lethal means (n=135, 15.9%), exposure to trauma (n=41, 4.8%), and public policies or other government actions (eg, anti-LGBTQ+ laws and ending no-fault divorce laws; n=41, 4.8%; Table 2). Among the 135 posts that identified lethal means as a cause, firearms were the most frequently identified method (n=111, 82.1%), followed by poisoning (n=13, 9.3%), and jumping (n=10, 7.1%). Firearms were identified as a cause of suicide significantly more frequently among Democrats (115/573, 20.1%) than Republicans (20/268, 7.5%; *P*<.001). Democrats were also significantly more likely than Republicans to identify causes related to barriers to accessing mental health services (17/573, 3% vs 2/268, 0.7%; *P*=.047), public policies or other government actions (34/573, 5.9% vs 7/268, 2.6%; *P*=.04), and any lethal means (115/573, 20.1% vs 20/268, 7.5%; *P*<.001).

In contrast, Republicans were significantly more likely than Democrats to identify social media (9/268, 3.4% vs 5/573, 0.9%; *P*=.009) and substance use or addiction (7/268, 2.6% vs 4/573, 0.7%; *P*=.04) as a cause of suicide. Republicans were also significantly more likely identify gender-affirming care as a cause of suicide than Democrats (30/268, 12.6% vs 1/573, 0.2%; *P*<.001).

**Table 2.** Causes of suicide identified in state legislators’ X posts mentioning suicide from December 2023 to November 2024^a^.

	Full sample (n=849), n (%)	Democrat posts (n=573), n (%)	Republican posts (n=268), n (%)	Chi-square (*df*)	*P* value
Any cause of suicide identified				0.449 (1)	.50
No	534 (62.9)	354 (61.8)	172 (64.2)		
Yes	315 (37.1)	219 (38.2)	96 (35.8)		
Causes identified					
Barriers to accessing treatment or services	19 (2.2)	17(3.0)	2 (0.7)	4.077 (1)^b^	*.*04
Chronic stressors	10 (1.2)	6 (1.0)	4 (1.5)	—^c^	.73
Explicit policy or government actions	41 (4.8)	34 (5.9)	7 (2.6)	4.345 (1)	.04
Exposure to trauma	41 (4.8)	32 (5.6)	9 (3.4)	1.952 (1)	.16
Lethal means or method of suicide	135 (15.9)	115 (20.1)	20 (7.5)	21.537 (1)	<.001
Mental illness	23 (2.7)	12 (2.1)	11 (4.1)	2.774 (1)	.01
Social media	14 (1.6)	5 (0.9)	9 (3.4)	—	.02
Stigma related to suicide or help seeking or treatment seeking	24 (2.8)	19 (3.3)	5 (1.9)	1.385 (1)	.24
Substance use or addiction	11 (1.3)	4 (0.7)	7 (2.6)	—	.04
Gender-affirming care	31 (3.7)	1 (0.2)	30 (12.6)	62.45 (1)	<.001
Antitransgender sentiments	3 (0.4)	3 (0.5)	0 (0)	—	.57
Encouraging suicide	5 (0.6)	2 (0.3)	3 (1.1)	—	.33

a Eight X posts were excluded from the comparisons between Democrats and Republicans because they were posted by Independents, a group too small to analyze independently.

b No test statistic value because reporting Fisher exact test

c Not applicable.

### Solutions Identified to Address Suicide

Of 849 posts, 558 (65.7%) identified at least one solution to prevent suicide, with Democrats being significantly more likely to identify a solution than Republicans (443/573, 77.3% vs 114/268, 42.5%; *P*<.001). The most frequently identified solutions were education or public awareness campaigns (eg, recognizing World Mental Health Day; n=299, 35.2%), policy changes (n=198, 23.3%), the 988 Suicide and Crisis Lifeline (n=175, 20.6%), help seeking not explicitly related to 988 (n=169, 19.9%), and restricting access to lethal means (n=105, 12.4%; Table 3).

Democrats were significantly more likely than Republicans to identify the 988 Suicide and Crisis Lifeline (140/573, 24.4% vs 34/268, 12.7%; *P*<.001) as well as other crisis lines (34/573, 5.9% vs 2/268, 0.7%; *P*<.001). Democrats were also significantly more likely to identify policy changes as a solution (149/573, 26% vs 49/268, 18.3%; *P*=.01) as well as restricting access to lethal means (93/573, 16.2% vs 12/268, 4.5%; *P*<.001).

**Table 3.** Solutions to address suicide identified in state legislators’ X posts mentioning suicide from December 2023 to November 2024^a^.

	Full sample (n=849), n (%)	Democrat posts (n=573), n (%)	Republican posts (n=268), n (%)	Chi-square (*df*)	*P* value
Any solution to address suicide mentioned				98.730 (1)	*<.*001
No	291 (34.3	130 (22.7)	154 (57.5)		
Yes	558 (65.7)	443 (77.3)	114 (42.5)		
Solution identified					
Clinical services and interventions	57 (6.7)	45 (7.9)	12 (4.5)	3.293 (1)	.07
Encouraging help seeking	169 (19.9)	133 (23.2)	35 (13.1)	11.771 (1)	<.001
Education or public awareness campaigns	299 (35.2)	234 (40.8)	64 (23.4)	22.949 (1)	<.001
Hotlines other than 988	36 (4.2)	34 (5.9)	2 (0.7)	11.992 (1)	<.001
Policy in general	198 (23.3)	149 (26)	49 (18.3)	6.046 (1)	.01
Reducing exposure to traumatic events	6 (0.7)	5 (0.9)	1 (0.4)	—^b^	.43
Restricting access to lethal means	105 (12.4)	93 (16.2)	12 (4.5)	23.083 (1)	<.001
988 Suicide and Crisis Lifeline	175 (20.6)	140 (24.4)	34 (12.7)	15.354 (1)	<.001

a Eight X posts were excluded from the comparisons between Democrats and Republicans because they were posted by Independents, a group too small to analyze independently.

b No test statistic value because reporting Fisher exact test.

### Identified Collateral Consequences of Suicide

Collateral consequences of suicide were only identified in 1.4% (12/849) of posts, with bereavement being identified as the consequence in most (9/12, 75%) of these posts. There was no significant difference in frequency of collateral consequences being identified between Democrats (8/573, 1.4%) and Republicans (4/268, 1.5%; *P*=.91).

### Guideline Nonconcordant Use of the Word Suicide

Of 849 posts, 78 (9.2%) used the word suicide in a way that was nonconcordant with the SAVE guidelines. Guideline nonconcordant posts were significantly less frequent in the posts of Democrats than Republicans (16/573, 2.8% vs 57/268, 21.3%; *P*<.001).

## Discussion

### Principal Findings

This study characterized the frequency and content of state legislators’ X posts mentioning suicide between December 1, 2023, and November 30, 2024. Consistent with research showing that suicide is the second leading cause of death among young people, youth were the most frequently identified population [29]. Other high-concern groups, such as veterans, firearm owners, and LGBTQ+ individuals, were also frequently identified. Populations with disproportionately high suicide rates, such as older adults and first responders [30-33], were either mentioned infrequently or not mentioned at all. This finding highlights a need to better educate state legislators about the epidemiology of suicide among these high-risk populations.

Legislators’ X posts mentioned solutions to address suicide more frequently than causes of suicide, which is consistent with the fact that legislators have the ability to address social problems via policymaking. Two-thirds of posts identified solutions to address suicide, with Democrats being significantly more likely to identify a solution than Republicans. This partisan difference was also observed for specific solutions, such as policy changes, implementation of the 988 Lifeline, and restricting access to lethal means. The most common solutions identified that shared bipartisan support were education or public awareness campaigns and help seeking in general.

For both Democrats and Republicans, the most frequent type of post was one recognizing an awareness campaign such as National Suicide Month or World Mental Health Day. While there are potential benefits to state legislators posting around a specific awareness day or month, such actions are more symbolic than substantive and do not reflect the power that state legislators have to help address risk factors for suicide [34-36]. Critically, state legislators’ ability to help prevent suicide lies in their ability to introduce and vote in favor of bills that address proximal risk factors for suicide and preventive interventions, such as limiting access to lethal means, improving access to mental health care, and increasing funding for the 988 Suicide and Crisis Lifeline [4]. Additionally, as was identified in a 2025 systematic review, there are many public policies that can help affect distal, structural determinants of suicide risk (eg, by reducing economic stress through increases in the minimum wage rate) [4]. These types of policies were rarely or never mentioned in legislators’ posts mentioning suicide. This suggests a need for improved dissemination of evidence to policymakers about the range of policies that have the potential to prevent suicide.

Democrats posted significantly more than Republicans about firearm owners or people with easy access to firearms, as well as lethal means restriction as a solution to prevent suicide. This is consistent with evidence indicating that Republicans are generally stronger supporters of the Second Amendment and have higher rates of firearm ownership than Democrats [37], and it follows that Republican legislators would be less focused on firearm ownership and access as they pertain to suicide. Suicide prevention dissemination campaigns may benefit from tailoring their language to Republican legislators and voters by focusing on evidence related to firearms and suicide. Democrats’ posts, however, are more consistent with research evidence, which suggests the potential importance of lethal means restriction and safe storage [4].

Nearly 10% (78/849) of posts used the word suicide in a way that was nonconcordant with the guidelines of SAVE. This finding suggests that policymakers would benefit from consultations with organizations such as the Suicide Prevention Resource Center or the American Foundation for Suicide Prevention to improve their use of appropriate and responsible suicide-related language.

### Limitations

This study is the first to examine state legislators’ social media posts mentioning suicide. However, the study has some limitations. All posts analyzed were from the period after Elon Musk purchased X, and it is possible that some legislators, especially Democrats, left the platform due to this change in leadership. Additionally, because not all state legislators are on social media, let alone X, the study is not a completely comprehensive assessment of how state legislators publicly discuss suicide. Furthermore, state legislators’ themselves may not be the ones posting social media content and may instead delegate posting responsibilities to social media management legislative aides who write and post the messages.

### Conclusions

This is the first study to document the frequency and content of state legislators’ social media posts about suicide. When considered within the context of research about the epidemiology of suicide and evidence for policies that can prevent suicide, this study signals a need to improve communication pertaining to suicide to state legislators and encourage collaboration with suicide prevention organizations and researchers. Furthermore, given differences observed between Democrat and Republican legislators, the study findings suggest potential value in tailoring messages about suicide for legislators based on their political party affiliation.

## Supplementary material

10.2196/83018 Multimedia Appendix 1 Codebook.

## Abbreviations

LGBTQ+: lesbian, gay, bisexual, transgender, queer, and more
