# Supplementary material for: Prevalence, Themes, and Partisan Differences in US State Legislator X Posts Mentioning Suicide: Content Analysis
Source: JMIR Form Res. 2026 May 25;10:e83018. doi: 10.2196/83018 (PMC13200803; doi:10.2196/83018)
Supplement: Multimedia Appendix 1 [file formative-v10-e83018-s001.docx]

Coding Guide for “Legislators Tweets Related to Suicide”

| **Code** | **Definition** |
| --- | --- |
| **Causes** | Tweet discusses what influences suicide, why people die by suicide, other mental illnesses and how they influence suicide |
| No |  |
| Yes | If yes, will have list of causes |
| **Solutions** | Specific laws or prevention guidelines, education plans, lethal means restriction, interventions |
| No |  |
| Yes | If yes, list solutions |
| **Collateral Consequences** | Bereavement, loss productivity in workforce etc. |
| No |  |
| Yes | If yes, list collateral consequences |
| **Derogatory** | Uses the word suicide or related terms in a joking manner, makes light of suicide |
| **“Generic Statements”** | Suicide mentioned but no content (does not include explicit mention of awareness) |
| **Awareness** |  |
| **Population** | Mentions a specific population |
| *Subcodes* |  |
| Kids/Youth |  |
| LGBTQ+ |  |
| Law Enforcement |  |
| Veterans |  |
| Other |  |
| **Medically assisted/euthanasia** | Mentions suicide in relation to medically assisted suicide or euthanasia |
| *Subcodes* |  |
| Positive | Speaks about medically assisted suicide or euthanasia positively |
| Negative | Speaks about medically assisted suicide or euthanasia positively |
| **Political View** |  |
| *Subcodes* |  |
| Democrat |  |
| Republican |  |
| Independent |  |
| **State** | Code what state the lawmaker is from |
| **Did they follow the safe reporting guidelines for media?**  **(https://www.save.org/media/media-recommendations/)** |  |
| *Used inappropriate language* | “Certain phrases and words can further stigmatize suicide, spread myths, and undermine suicide prevention objectives such as “committed suicide” or referring to suicide as “successful,” unsuccessful” or a “failed attempt.” Instead use, “died by suicide” or “killed him/herself.”’ |
| No |  |
| Yes | If yes, code what the inappropriate language is |
